# Survival of total knee arthroplasty in patients with Parkinson’s disease: a registry study

**DOI:** 10.1007/s00264-025-06658-2

**Published:** 2025-09-25

**Authors:** Alessandro Panciera, Alberto Di Martino, Barbara Bordini, Marina Amabile, Claudio D’Agostino, Vitantonio Digennaro, Cesare Faldini

**Affiliations:** 1 1st Orthopardic Department, IRCCS - Istituto Ortopedico Rizzoli, Bologna, Italy; 2 Department of Biomedical and Neurimotor Science - DIBINEM - University of Bologna, Bologna, Italy; 3 Medical Technology Laboratory, IRCCS - Istituto Ortopedico Rizzoli, Bologna, Italy

**Keywords:** Total Knee Arthroplasty, Parkinson, Knee, Survivorship, Registry

## Abstract

**Purpose:**

This study compared the demographics and outcomes of patients with Parkinson’s disease (PD) undergoing total knee arthroplasty (TKA) to those without PD. Additionally, it aimed to assess the impact of implant design on TKA survival in PD patients.

**Methods:**

Using data from the Emilia Romagna Registry of Orthopedic Prosthetic Implants, 551 TKA procedures in patients with PD were identified and compared to 52,022 TKAs in patients without PD. Kaplan-Meier survivorship analysis was used to compare implant survival, with revision surgery as the endpoint. Cox multivariate analysis was performed to assess the influence of age, gender, PD diagnosis, and implant design on implant failure.

**Results:**

The average age of PD patients was 72.2 years, with 66.2% being female. Implant survival was significantly lower in the PD group compared to the control group (*p* < 0.001). At 13 years, the survival rate was 88.8% in the PD group and 94.3% in the control group. PD patients had a 2.7 times higher risk of implant failure after adjusting for age and gender. Constrained implant designs were associated with a 1.7 times higher risk of failure compared to non-constrained designs in PD patients.

**Conclusion:**

PD negatively affects implant survival in patients undergoing TKA. Careful consideration should be given to patient selection and implant design in this patient population.

## Introduction

Total knee arthroplasty (TKA) represents one of the most common elective orthopaedic procedures performed worldwide, and the number of procedures is expected to increase in the coming years [1]. This increment is led by the development of broader indications for TKA, including patients that are afflicted with more complex conditions such as neurological disorders.

Among neurological disorders, Parkinson’s disease (PD) is a frequently identified risk factor for prosthetic implant failure, leading to revision surgery [2]. PD is the second most common neurodegenerative disorder after Alzheimer’s disease (AD), but the most common with musculoskeletal symptoms. PD is characterized by the loss of dopaminergic neurons in the pars compacta of the substantia nigra and has a slow progression associated with a wide variety of symptoms, ranging from autonomic nervous system disorders to sensory abnormalities to motor symptoms. Motor symptoms include tremors, bradykinesia, rigid contractures, dystonia, and a flexed posture. The onset of symptoms is usually asymmetrical and progressively can involve the entire patient hemisoma.

The orthopaedic management of PD can be extremely challenging, due to the severity of gait abnormalities, postural instability, and limited exercise capacity of these patients [3]. Moreover, PD patients often present an involvement in other joints, specifically the hip and the spine, which can alter the normal biomechanics of the knee. Typically, PD patients present with a spine sagittal imbalance compensated through pelvic retroversion, hip and knee flexion. This stance, along with muscular rigidity and weakness, poor coordination, and unsteadiness, contributes to the early onset of osteoarthritis (OA). Moreover, these symptoms can alter the biomechanics of an implant, leading to mechanical complications such as implant loosening and dislocation. Therefore, in patients suffering from PD TKA can be a difficult procedure that involves multiple specialists to ensure the best possible results [4], but such outcomes can be very unpredictable [5].

This paper aims to: (1) compare features and differences of PD patients with a control group; (2) analyze TKA survival in PD patients versus the control group and determine implant failure risk; (3) investigate whether different constraint designs affect TKA longevity in PD patients.

## Materials and methods

The study was conducted through the gathering and examination of data from the Emilia Romagna Registry of Orthopedic Prosthetic Implants (RIPO), which is a comprehensive database founded by the Rizzoli Orthopedic Institute in 1990, documenting primary and revision procedures for hip, knee, and shoulder replacements. This registry has been continuously updated by all 68 Orthopedic Departments of the regional territory of Emilia-Romagna since January 2000 and allows for comparisons with other National Registries. Additionally, our healthcare organization had access to administrative databases including hospital discharge summaries, outpatient services, medication prescriptions, and healthcare cost waivers. Using these tools and combining it with the RIPO data we were able to identify patients suffering from PD that underwent total knee arthroplasty between 01/01/2003 and 31/12/2018 in the Emilia-Romagna region. To identify these patients the databases were searched with a specific algorithm: patients had to have at least one hospitalization with a primary or secondary diagnosis of PD, identified by the ICD-9 codes 332.0, 332.1, 333.0 ore 781 before the TKA surgery, as well as at least two drug prescriptions for PD, identified by the ATC N04* code in the year before the TKA. Patients that had a PD diagnosis made after the TKA procedure were excluded.

Considering the inclusion and exclusion criteria, the RIPO registry provided data on 52,573 TKA procedures carried out during the specified period. Procedures performed on patients living outside Emilia-Romagna region were excluded, to minimize bias due to loss at follow-up. This is because patients living in other regions, which performed primary surgery in Emilia-Romagna, but an eventual revision outside would not be identified by the RIPO, and therefore survival data would be biased. Utilizing our search algorithm, we detected 52,022 TKAs (99.0%) performed on patients without a PD diagnosis and 551 TKAs (1.0%) on patients with a PD diagnosis. Out of the 551 PD patients who underwent TKA, 276 cases were identified solely based on their prescription medications, 157 cases were detected using ICD-9 codes, and 118 cases met both identification criteria.

### Statistical analysis

Descriptive statistics, such as median and range for continuous variables and frequency with percentage (%) for categorical variables were used for data report. The Chi-square test was employed to assess statistical significance of qualitative data, while the t-test was used for continuous data. Kaplan-Meier survivorship analysis was performed using the revision as endpoint, with implant survival of non-revised THAs considered as the last date of observation (December 31st, 2019, or the date of death available from the ER database). The Wilcoxon test was used to compare survivorship between groups. The Wald test was conducted to analyze the *p* values for data achieved from the Cox multiple regression analyses. The proportional hazards assumption was estimated using the Schoenfeld residual method and *p* values < 0.05 were considered significant. Statistical analyses were conducted using JMP, version 12.0.1 (SAS Institute Inc., Cary, NC, USA, 1989–2007).

## Results

According to our data, 551 implants were performed on 491 patients diagnosed with PD, whereas 52,022 implants were performed on 43,304 patients without PD. The distribution between right and left sides showed no significant difference: for the PD group, 311 implants (56.4%) were right-sided, while in the control group, 27,600 implants (53.1%) were right-sided. A significant difference (*p*-value 0.01) in gender distribution was observed between the two groups: within the PD group, 365 cases (66.2%) were female, contrasted with 36,950 female patients (71.0%) in the control group. This observation is likely attributable to the disproportionately high prevalence of Parkinson’s disease among males, which consequently reduces their representation in the control group. The mean age of patients at the time of surgery was 72.2 years (range 42–89) in the PD group compared to 71.4 years (range 21–95) in the control group; this age difference was statistically significant as determined by a t-test (*p*-value 0,02). There was no statistically significant difference observed between the PD group and the control group in terms of tibial insert mobility (*p*-value 0.34), insert material (*p*-value 0.21), and femoral component material (*p*-value 0.82) (Table 1).

**Table 1:** Differences between the PD group and the control group. Only the differences in gender and average age where significantly different, whereas etiology, bearing characteristics e the material of the implant were not significant

	PD group	Control group	
***N° of implants (%)***	551 (1,0%)	52.022 (99,0%)	
***N° of patients***	491	43.304	
**Side**			
DX (%)	311 (56,4%)	27.600 (53,1%)	
***Gender***			
Females (%)	365 (66,2%)	36.950 (71,0%)	Fisher’s exact test (*p* = 0,01)
***Average age (range)***	72,2 (42–89)	71,4 (21–95)	Student’s t test (*p* = 0,02)
***Etiology***			
Primary OA (%) Deformities (%)	82,6 10,8	84,4 9,8	Pearson’s Chi-squared test (*p* = 0,5)
***Mobility of the bearing***			
Fixed (%)	68,9	66,9	Fisher’s exact test (*p* = 0,34)
***Material of the bearing***			
Convention poly (%) Cross-linked poly (%) Cross-lnk poly + vit E (%)	82,4 13,4 4,2	83,3 11,4 5,3	Pearson’s Chi-squared test (*p* = 0,21)
***Material of the prosthesis***			
CrCo(%) Oxinium (%) Ceramized CrCo (%) Other (%)	88,8 6,9 3,5 0,7	88,4 7,4 3,7 0,5	Pearson’s Chi-squared test (*p* = 0,82)

The Kaplan-Meier method was employed to calculate the survival rate of the TKA. The X axis denotes time in years, while the Y axis represents the percentage of surviving implants. The endpoint was identified as being the revision of the tibial insert and/or the femoral component and/or of the tibial component. Secondary patellar substitution was not considered as an implant failure in this case. Implant failures were documented until 31/12/2018. In detail, within the PD group, out of 551 TKAs monitored over an average period of 5.2 years, there were 46 revisions noted. Conversely, in the control group, from a total of 52,022 TKAs with an average follow-up of 6.3 years, 1,823 revisions were recorded (Table 2).

**Table 2:** Results comparing the revision incidence between PD patients and the control group showed a significant difference

Groups	TKAs	Average follow-up (range)	TKA revisions	Revision incidence (*n*° revision/*n*° TKAs)
PD group	551	5,2 (0–15,9 years)	46	8,4%
Control group	52.022	6,3 (0–16,0 years)	1.823	3,5%

**Fig. 1:**
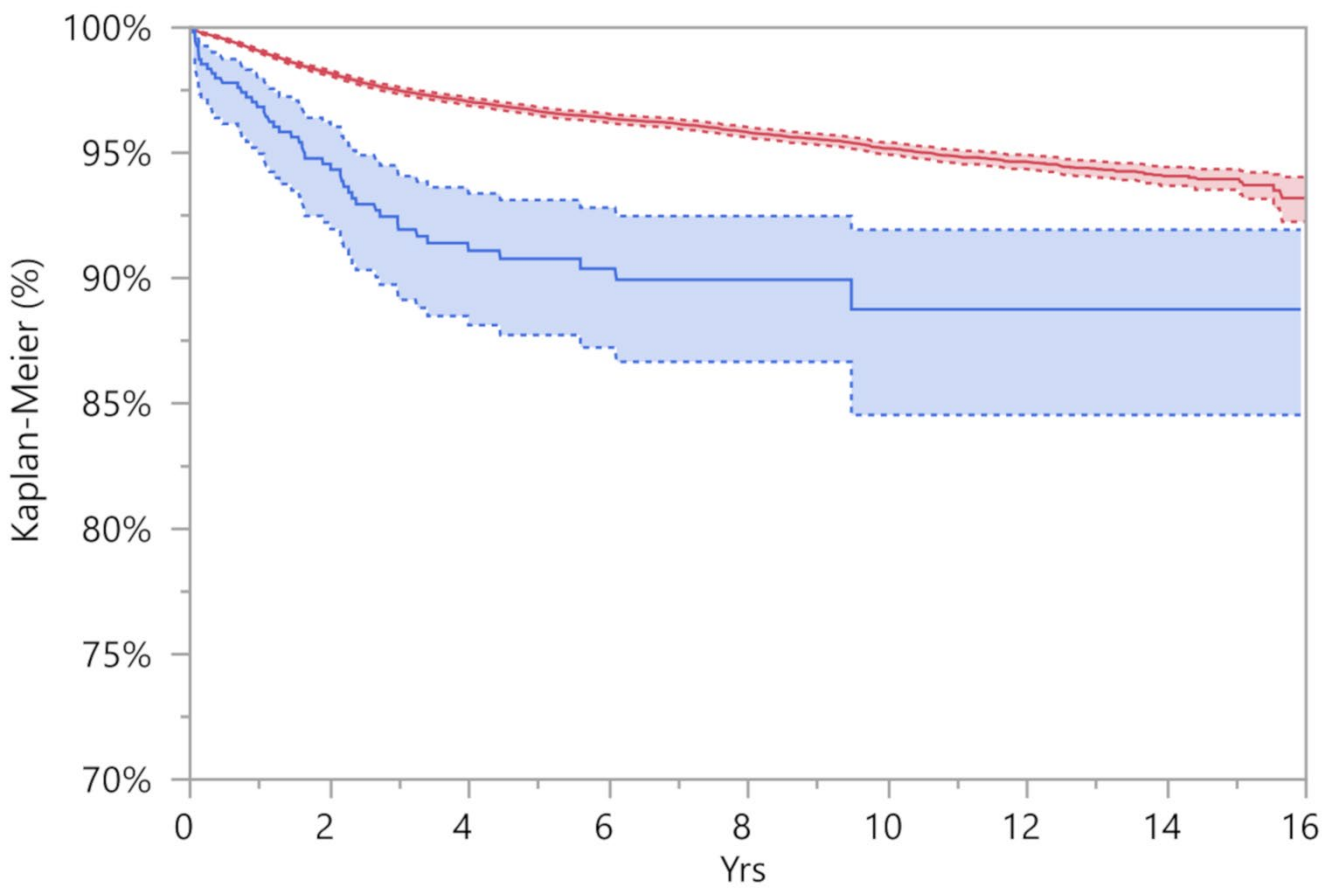
the Kaplan-Meier curves showed significant differences between the PD group (blue line) and the control group (red line). The dotted lines represent the confidence interval of 95%

The reported findings indicate a significant difference between the two Kaplan-Meier curves, as evidenced by a *p*-value of < 0.001 in the Wilcoxon test (Fig. 1). As it is represented in Fig. 1 the survival rate in PD patients appears to be significantly lower than in the control group.

We further explored differences in the survival rate between the two groups, delving more in detail at various times of follow-up. The difference in survival rate starts to be significant early, and is visible at one year of follow-up, only increasing over time (Table 3). At the one year follow-up, the survival rate in the Parkinson’s disease (PD) group was 96.8%, compared to 99.1% in the control group. This difference remained significant over time, with survival rates at 13 years of 88.8% and 94.3% for the PD and control groups, respectively. As shown in Table 3, the at-risk population gradually decreased over time due to the reduced number of patients with longer follow-up periods.

**Table 3:** Results comparing the survival rate at various follow-up

Survival rate % (Confidence Interval 95%)						
Group	1 year	3 years	5 years	7 years	10 years	13 years
PD group	96,8 (95,0–98,0)	91,9 (89,1–94,1)	90,8 (87,7–93,1)	89,9 (86,7–92,5)	88,8 (84,5–91,9)	88,8 (84,5–91,9)
*At-risk population*	497	355	254	172	*62*	*19*
Control group	99,1 (99,0–99,1)	97,5 (97,3–97,6)	96,7 (96,5–96,8)	96,1 (96,0–96,3)	95,2 (94,9–95,4)	94,3 (94,0–94,7)
*At-risk population*	47.176	37.737	29.446	21.659	*11.055*	*3.604*

As shown in Table 4, we investigated the causes underlying the implant failures. Our findings include both the occurrence rates of the different etiologies and their percentage distribution. We considered the following causes for implant failure: aseptic loosening (including isolated tibial or femoral loosening), septic loosening, pain without loosening, implant dislocation, periprosthetic fracture, implant instability, polyethylene wear, and joint stiffness. As reported in Table 4, patients with parkinson’s disease (PD) had an increased risk of septic loosening and implant dislocations. The incidence of other causes was similar between the PD and control groups.

**Table 4:** Causes of implant failure and their respective incidence and percentage distribution. The causes are reported in order based on their incidence in the control group

Cause of implant failure	Control Group			PD Group		
	Incidence	%	Percentage distribution	Incidence	%	Percentage distribution
Aseptic loosening	490/52.022	0,9	26,9	12/551	2,2	26,1
Septic loosening	460/52.022	0,9	25,2	16/551	2,9	34,8
Pain w/out loosening	186/52.022	0,4	10,2	3/551	0,5	6,5
Tibial aseptic loosening	166/52.022	0,3	9,1	4/551	0,7	8,7
Cause not reported	205/52.022	0,4	11,2	3/551	0,5	6,5
Other	63/52.022	0,1	3,5	4/551	0,7	8,7
Implant dislocation	57/52.022	0,1	3,1	3/551	0,5	6,5
Periprosthetic fracture	43/52.022	0,1	2,4	1/551	0,2	2,2
Implant instability	42/52.022	0,1	2,3	-	-	-
Polyethylene wear	35/52.022	0,1	1,9	-	-	-
Femoral aseptic loosening	33/52.022	0,1	1,8	-	-	-
Joint stiffness	28/52.022	0,1	1,5	-	-	-
Implant rupture	15/52.022	0,03	0,8	-	-	-
Total	1.823/52.022	3,5	100,0	46/551	8,3	100,0

Finally, we performed a Cox multivariate analysis on our set of data. The Cox multivariate analysis identifies any variables that are independent of each other that can influence the event, in our case implant failure. Analysis was performed on four independent variables: gender, age at surgery, PD diagnosis (PD group vs. control group), and implant design. The proportionality hazard was tested by the Schoenfeld residual method. All four variables included in the model are statistically significant, namely age at surgery (*p* < 0.0001), gender (*p* < 0.0001), PD diagnosis (*p* = 0.0348), and implant design (*p* < 0.0001) with the following hazard ratios (HR):

- HR PD group vs. control group = 2,7 (95% CI 2,1–3,7). It namely means that patients with PD have a 2,7 times higher risk than patients in the control group, considering the same age and gender.
- HR age ≤ 60 years vs. age > 60 years = 2,2 (95%CI 1,9 − 2,5). It means that patients under 60 years of age have a 2,2 times higher risk, considering the same gender and PD diagnosis.
- HR gender M/F = 1,3 (95%CI 1.1–1.4). It means that male patients have a higher risk, specifically 1,3 times higher, considering the same age and PD diagnosis.
- HR implant design (constrained vs. non-constrained implants) = 1,7 (95%CI 1.3–2.1). It means that patients with constrained implants (semi-constrained and rotating hinge implants) have a risk 1,7 times higher considering the same age and gender when compared to patients with non-constrained implant designs.

## Discussion

### Summary of results

Our research analyzed 551 total knee arthroplasties (TKAs) conducted on patients with Parkinson’s disease (PD) from January 1, 2003, to December 31, 2018. Among these patients, 66.2% (365) were women, which is a smaller proportion compared to the control group (71.0%), suggesting a higher prevalence of PD among male patients. The average age at the time of surgery was 72.2 years (range 42–89) in the PD group compared to 71.4 years (range 21–95) in the control group. There was no statistically significant difference observed between the PD group and the control group in terms of tibial insert mobility (*p*-value 0.34), insert material (*p*-value 0.21), and femoral component material (*p*-value 0.82).

A comparison of implant survival between the PD group and the control group revealed a significant difference. Specifically, among 551 TKAs performed in PD patients we reported 46 (8,4%) revisions with an average follow-up of 5.2 years (range 0–15,9 years), while in the control group among 52,022 TKAs performed we reported 1823 (3,5%) revisions with an average follow-up of 6.3 years (range 0–16 years). The PD group was found to be 2,8 times more likely to experience implant failure than the age- and gender-matched control group.

### Limitation of the study

Our study has several limitations. Firstly, it relies on a registry and uses an algorithm with specific inclusion and exclusion criteria. Yet, due to challenges in defining and diagnosing PD, as well as issues with data collection and information flow, the prevalence of PD might be underestimated. Moreover, another substantial limitation is the retrospective design, which relied on observational data; hence, it was not feasible to establish cause-effect relationships or assess individual confounding factors. Finally, data concerning the pre-operative severity of PD, medical therapy and functional outcomes were not available to be studied.

### Discussion of the results

The likelihood of negative outcomes following TKA procedures is higher in patients suffering from neurological conditions. PD represents one of the most common, yet most complex, neurological disorders with primarily motor symptoms. Therefore, it is one of the most studied neurological disorders and while there is still no cure, with proper treatment patients usually have a life expectancy exceeding tenyears after the PD diagnosis. These patients can easily develop musculoskeletal disorders associated with age such as knee OA. While much has been written about primary TKA and its outcomes, far less has been written about TKAs performed in patients suffering from PD. No clear guidelines are available to aid the surgeon in determining the indication for surgery or to guide them during the surgical procedure to optimize the outcomes in this subcategory of patients.

The aim of this paper is to provide an accurate and relevant description of the extent of increased risk of prosthetic implant failure in Parkinson patients, and specifically: (1) assessing the characteristics and relative differences between the PD patients and the control group; (2) evaluating the survival rate of TKA in PD patients compared to controls and the relative risk of implant failure.

#### Question 1

Concerning the differences between the two groups, as reported in Table 1, the only significant differences between the PD group and control groups were found in gender and age. Gender represents a known risk factor for PD, specifically males are diagnosed with a ratio of 2:1 when compared to females [6–8]. Furthermore, the clinical manifestation of Parkinson’s Disease (PD) varies between genders. According to Haaxma et al. [9], females tend to be older than males at onset (by an average of 2.1 years) and are more likely to exhibit tremors as their initial symptom. In contrast, males typically present with either bradykinesia or rigidity as their first symptom. These differences explain why our PD group has fewer female patients compared to our control group, specifically 66,2% vs. 71,0% respectively. Nonetheless, women constitute the majority of patients, as symptomatic knee osteoarthritis is more prevalent in females than in males. This disparity can be attributed to various hormonal, anatomical, and biomechanical factors [10].

Age represents another significant difference between our two groups. Specifically, the PD group presents an average age of 72,2 years while the control group an average age of 71,4 years. This increased average age can be correlated to the higher incidence of PD in older patients: specifically, PD incidence peaks at 80 years of age [2, 11]. This difference is not so clearly found in the available literature since the difference between these two groups if often reported as not significant [2].

We also investigated the aetiology of the condition, bearing mobility and material, and prosthesis material in both groups. We found no significant differences between the two study groups in these aspects. Specifically, regarding OA aetiology, we excluded numerous cases of secondary knee OA that could act as confounding factors, focusing solely on primary OA and secondary OA resulting from deformities. In the PD group, primary OA accounted for 82.6% of cases, while OA due to deformities represented 10.8%. In the control group, the corresponding percentages were 84.4% and 9.8%, respectively. The differences were reported as not significant using di Pearson’s Chi-squared test.

We also observed no significant difference in polyethylene bearing material and mobility between the groups. However, our results regarding material suggest a potential issue with data collection. The percentages of “modern” cross-linked polyethylene, which has largely replaced conventional polyethylene, seem underrepresented. We attribute this possible bias to how polyethylene bearings are packaged and labeled. If the production method is not specified, the registry classifies the polyethylene as conventional.

#### Question 2

Total knee arthroplasty has been shown to be an effective treatment for knee OA in PD patients, leading to significant improvements in functional scores. While Yoon et al. [2] reported that TKA significantly improved both knee and functional scores, the degree of improvement in PD patients was less pronounced compared to those without PD [12, 13]. Furthermore, PD severity may influence the post-operative functional outcome. Rong et al. [12] reported that patients with mild PD experienced better clinical and functional results than those with more severe PD symptoms. However, Ergin et al. [14] found no significant difference in post-operative function between the two groups. Complications are believed to be more frequent in PD patients after TKA compared to non-affected patients. Five studies [15–19] report a higher complication rate in PD patients. Specifically, Marchand et al. [18] reported a 3.5 times higher rate of medical complications such as transfusions, anemia, cerebrovascular events, and thrombocytopenia. Additionally, they found that implant-related complications like loosening and periprosthetic fracture were 1.6 times more common in the PD group. However, Jamsen et al. [20] and Kleiner et al. [21] reported no differences between the complications rate between the two groups.

Nevertheless, no available literature is available regarding the survival rates between PD patients and a control group. As detailed in Table 3, significant differences emerge early on. Specifically, the survival rate for PD patients is 96.8% at one year of follow-up, decreasing to 88.8% at 13 years. In contrast, the control group exhibits a survival rate of 99.1% at one year, gradually declining to 94.3% at 13 years.

**Fig. 2:**
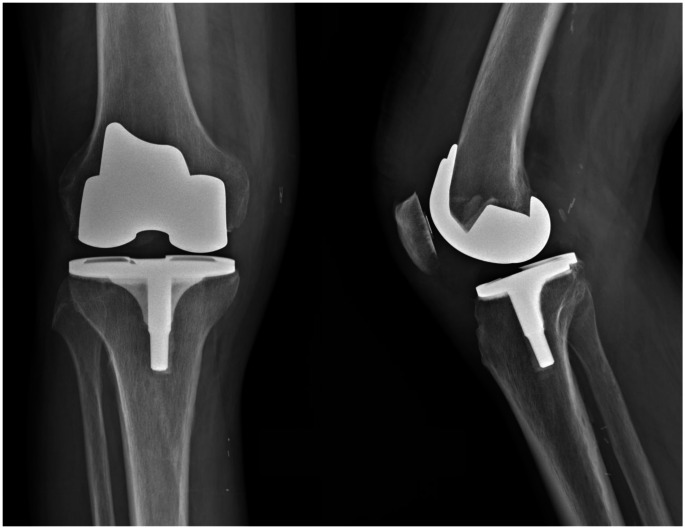
X-rays of a 79-year-old male individual with Parkinson’s disease, taken one year after surgery. The total knee arthroplasty (TKA) was performed using a functional alignment approach. At the follow-up, the patient demonstrated excellent clinical and functional outcomes, regaining full independence in daily activities. No signs of radiographic loosening of the implant can be found

**Fig. 3:**
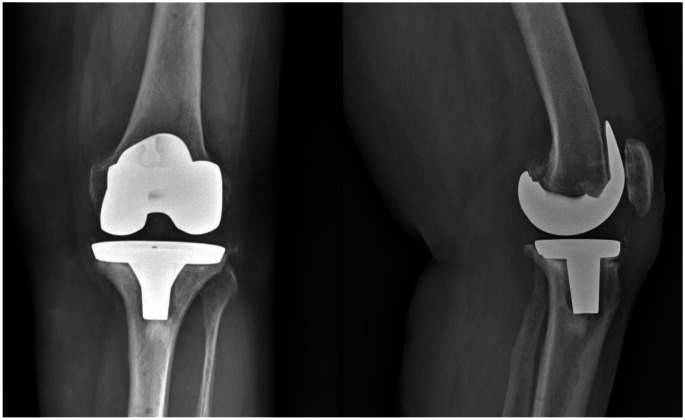
X-rays of a 72-year-old individual with Parkinson’s disease, taken one year following total knee arthroplasty (TKA) performed using a functional alignment technique. At the follow-up, the images revealed implant loosening, which was associated with poor functional and clinical outcomes and ultimately required a two-stage implant revision

As Table 4 indicates, the PD group experienced a higher risk of septic loosening compared to the control group (34.8% vs. 25.2%). This aligns with existing literature [17, 18] demonstrates an increased risk of periprosthetic joint infection (PJI) in patients with PD. However, the cause behind this heightened prevalence remains unclear. Marchand et al. [18] reported that even with this elevated PJI risk, revision TKA rates were not significantly higher in PD patients. They suggested that this discrepancy could be attributed to various factors, including advancing disease state, comorbidities, or patient mortality within the PD population. In our case, the increased risk of PJI also manifested in a higher rate of revisions, indicating that PJI is one of the most severe complications after TKA even in the PD population. As this is a registry-based study, we lack clinical data regarding the functional outcomes of these patients, since our primary variable of interest is implant survival. For this reason, it remains uncertain whether a longer implant survival can truly be considered a “good result”. Furthermore, our clinical experience reveals instances where patients—regardless of whether they have a PD diagnosis—display good post-operative radiographic alignment without signs of implant loosening and no clear indication for revision surgery yet still have disappointing functional outcomes (Figs. 2 and 3). In retrospective cohort studies, such cases would not be classified as successful results; however, registry studies categorize these patients as “survived,” thus reporting them as positive outcomes. This highlights a limitation of registry-based research, as it may not fully capture the true effectiveness of treatment in this patient population.

In conclusion, Parkinson’s disease is a challenging but relatively common condition that affects a large number of patients, some of whom may require total knee arthroplasty. Based on our data collected from the RIPO Registry, we found that TKAs in PD patients have a reduced survival rate at follow-up compared to the control group. Moreover, we found that the hazard ratio for implant failure in PD patients was 2.8 when adjusting for age and gender. These results suggest that PD patients represent a frail subgroup who may need specific treatment options to optimize their outcomes.

Further studies are needed to better understand how to optimally treat these patients and to explore specific prosthesis designs that may help achieve improved outcomes.

## Data Availability

No datasets were generated or analysed during the current study.
